# Formation of a traumatic air cyst and ensuing hemopneumothorax during CT angiography in a patient with Ehlers-Danlos syndrome

**DOI:** 10.1259/bjrcr.20200082

**Published:** 2020-07-29

**Authors:** Hans Michell, Prajna Chopra, Anant Bhave, Naiim Ali, William Parkinson, Joseph Shields, Geoffrey Scriver, Christopher Morris

**Affiliations:** 1 Section of Interventional Radiology, Department of Radiology, University of Vermont Medical Center, Burlington, Vermont, United States; 2 Department of Radiology, University of Vermont Medical Center, Burlington, Vermont, United States

## Abstract

Ehlers-Danlos syndrome (EDS) refers to a rare group of genetic disorders that makeup part of the connective tissue disorders consortium. It is characterized by clinical features such as skin hyperextensibility, joint hypermobility, and tissue fragility. A vascular subtype (EDS IV) exists, that predisposes affected patients to vascular injury and is well-known and documented. However, other manifestations of EDS IV are less commonly understood and reported. Though spontaneous pneumothorax has been described in several cases, formation of traumatic air cysts/pneumatoceles with little to no inciting factors has not. This can eventually lead to pulmonary hemorrhage or hemopneumothorax. We present a case of spontaneous formation of a traumatic air cyst with ensuing large-volume hemopneumothorax occurring in a time period of under 3 minutes, between pre- and post-contrast-media administration during CT angiography of the chest.

## Introduction

Ehlers-Danlos syndrome (EDS) refers to a rare group of genetic disorders that makeup part of the connective tissue disorders consortium. Characterized by clinical features such as skin hyperextensibility, joint hypermobility and tissue fragility, EDS has been classified into several different subtypes. The new international classification of EDS established in 2017 classifies EDS into 13 different subtypes, one of which includes the vascular subtype, Type IV.^1^ EDS Type IV (EDS IV) differs significantly from the other subtypes due to its potential life-threatening nature, secondary to the increased risk of the spontaneous vascular dissection and/or rupture.^2^ No curative treatment currently exists for EDS, and most of the current treatment options are either palliative or preventative. In acute emergencies, endovascular intervention for patients diagnosed with EDS IV can be considered, however, there is high-risk for vascular dissection or perforation due to predisposition to traumatic injury related to connective tissue fragility.

A rare manifestation of EDS IV is the intrathoracic formation of traumatic air cysts or pneumatoceles.^3–5^ The cysts predispose patients to pneumothorax, which can further deteriorate to hemopneumothorax. However, the true etiology of the underlying hemorrhage is unknown.^6^ Because Type III collagen is involved in synthesis of both blood vessel walls and pulmonary fibroblasts, the ensuing pulmonary hemorrhage may be secondary to a primary defect of the pulmonary parenchyma itself, or to intrapulmonary vascular rupture.^6^ Herein, we present a case of a patient with a known history of EDS IV who suffered a sudden large-volume hemopneumothorax that occurred between acquisition of pre-contrast media and post-contrast media injection at CT angiography. We propose this to have been secondary to a primary defect of the lung parenchyma (secondary to her EDS IV) as evidenced by the imaging findings further discussed below.

## Case

**Figure 1.**
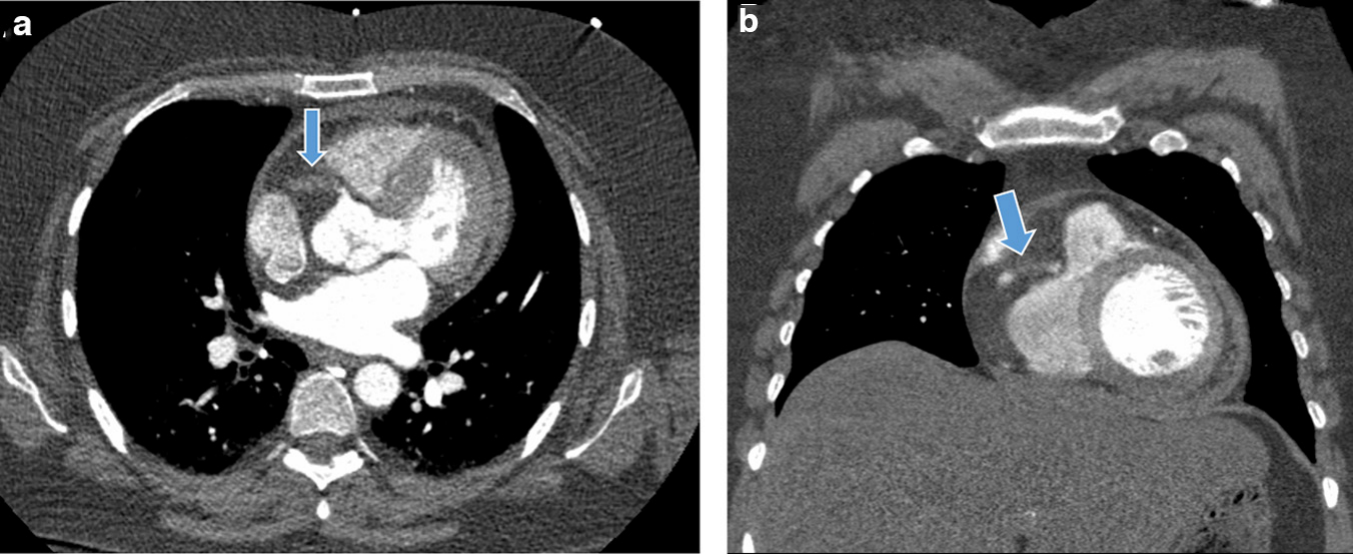
A and B. Contrast-enhanced CT images initially obtained for chest pain 5 days prior. (A.) Axial contrast-media-enhanced image of the chest demonstrating an abrupt 2 cm occlusion of the proximal right coronary artery. (B) Coronal reformat image better demonstrating the occluded segment of the right coronary artery. Normal contrast flow is seen just proximal and distal to this.

A 41-year-old female with a past medical history of migraines, hypertension, and EDS IV with multiple prior spontaneous vascular dissections (including superior mesenteric, splenic and bilateral iliac arteries) originally presented to the hospital with acute chest pain. CT angiography of the chest was performed and demonstrated occlusion of a 2 cm segment of the proximal right coronary artery (Figure 1A and B), which was likely due to dissection given her history and predisposition. Imaging also demonstrated a moderate-sized hemopericardium. During acquisition of the CT images, the patient suffered a cardiac arrest on the CT scanner table and a cardiopulmonary arrest code was initiated. The patient was found to be in ventricular fibrillation. Return of spontaneous circulation was achieved after 1 min of chest compressions and a single 200 Joule defibrillation. Cardiology work-up confirmed an acute ST elevation myocardial infarction (STEMI) complicated by pericardial hemorrhage likely secondary to the acute dissection. However, given her history of EDS IV, performing percutaneous coronary intervention was considered too high risk of a procedure. Conservative management was recommended with close clinical monitoring in the intensive care unit. A complete cardiologic work-up performed during her hospital stay found no further evidence of heart block or physiologic evidence of right ventricular infarction, and the patient suffered no further sinus arrest. The patient was monitored for two more days and discharged in stable condition.

**Figure 2.**
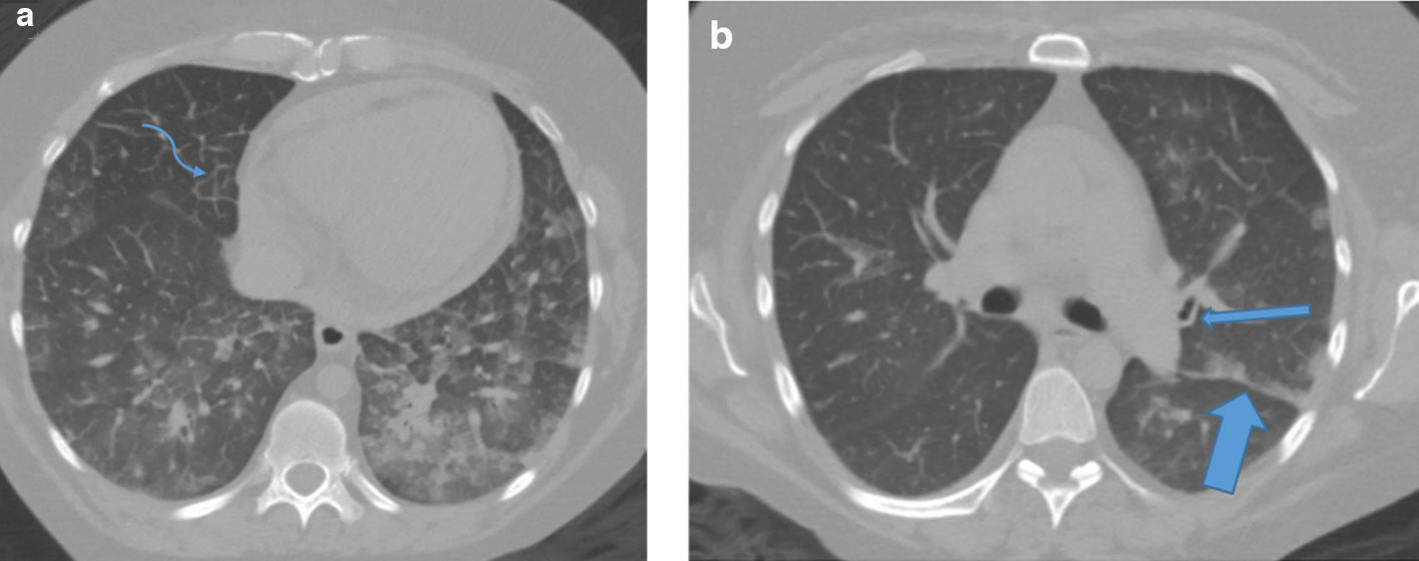
(A and B) Pre-contrast CT images imaging obtained for acute dyspnea on date of vascular injury. (A). Axial pre-contrast CT image of the chest at the level of the atrioventricular groove obtained immediately prior to angiography images demonstrating findings of pulmonary edema, including scattered ill-defined parenchymal groundglass opacities and interlobular septal thickening (curved arrow). (B). Axial pre-contrast CT image near the level of the carina demonstrating additional findings of pulmonary edema, including dependent groundglass opacities along the fissures (wide arrow), and peribronchial thickening (thin arrow).

**Figure 3.**
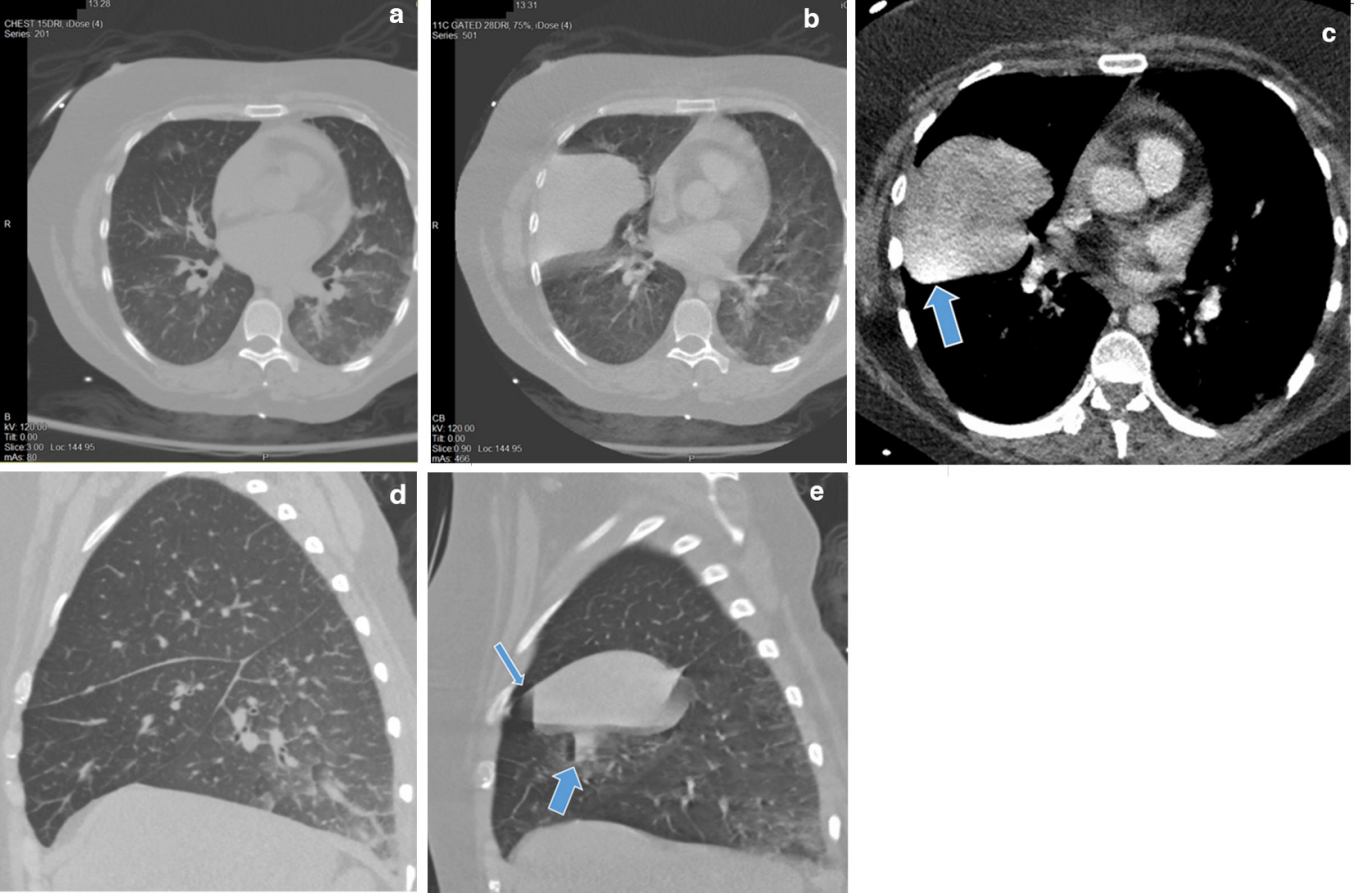
(A-E). CT angiography images obtained on date of injury. (A) Axial CT pre-contrast image at the level of the middle lobe showing no evidence of hemorrhage in this region. (B) CT angiography image obtained within 3 min of the pre-contrast imaging showing a new, large, dense collection filling the pleural space subjacent to the minor fissure. This was not present 3 min earlier on pre-contrast imaging (3A). Note the timestamp in the right upper corners of the images. (C) CT angiography image set to soft-tissue windowing better demonstrates a region of increased density at the most dependent portion of the collection with attenuation similar to contrast (arrow), confirming active extravasation of recently given contrast media. (D, E) Pre-contrast and angiograhy sagittal reformats better demonstrating lack of hemorrhage or pneumatocele/air cyst formation prior to contrast injection, with sudden spontaneous parenchymal hemorrhage on angiography imaging. Note the air-fluid level within the right middle lobe parenchyma (wide arrow) suggesting traumatic air cyst formation with immediate hematoma development. A second air-fluid level is seen in the pleural space (thin arrow) consistent with subsequent hemopneumothorax formation.

The patient returned to the emergency department 2 days later with acute shortness of breath. Initial assessment revealed hypoxia and increased respiratory effort. In concert with the patient’s physical exam, these findings were concerning for pulmonary edema secondary to acute myocardial infarction. A CT angiogram of the chest was obtained. The pre-contrast-media images confirmed the clinical suspicion of pulmonary edema (Figure 2A and B). The patient was then administered 100 cc of intravenous nonionic iodinated contrast media at 4 cc/s with imaging timed for peak systemic arterial enhancement. However, the angiogram images, which were obtained within 3 min of the pre-contrast-media images, demonstrated a new, large-volume, high attenuation fluid collection occupying a portion of the parenchyma of the middle lobe, with most of the fluid extending into the right minor fissure, findings consistent with spontaneous parenchymal hemorrhage (Figure 3A–C). Further interrogation demonstrated air-fluid levels within the middle lobe parenchyma and within the pleural space subjacent to the minor fissure, findings consistent with traumatic air cyst formation and hemopneumothorax, respectively (Figure 3D–E). The CT angiography was not optimized for opacification of the pulmonary arteries and, as such, a source of active extravasation was not visible.

Interventional radiology was consulted for possible embolization. However, as aforementioned, given the lack of suboptimal imaging parameters for the pulmonary arteries, thereby disallowing visibility of a possible target vessel, and the patient’s underlying EDS IV, conventional angiography and transcatheter embolization was not recommended. Subsequently, the patient and her family decided to pursue comfort care measures only. The patient ultimately expired later that evening due to cardiac arrhythmia secondary to tamponade from her hemopericardium.

## Discussion

This case demonstrates how truly life-threatening EDS IV can be. This patient suffered a sudden, large-volume hemopneumothorax within a time period of less than 3 min. As previously mentioned, the underlying cause of hemorrhage in cases of hemopneumothorax in EDS IV is unknown, likely owing to the paucity of reported cases on this entity. The two competing theories are fragility of the lung parenchyma, making it susceptible to injury with little to no inciting trauma, and the fragility of the intrapulmonary vasculature, making the vessels susceptible to rupture. In this case, we propose the former to have been the culprit as evidenced by the presence of spontaneous air cyst formation, which was not present on pre-angiography imaging. Furthermore, the patient underwent CT angiography imaging of the chest 5 days prior when her coronary artery dissection was diagnosed, and no CT evidence of vascular abnormality (*e.g.* aneurysm, pseudoaneurysm, dissection, etc.) was seen in this region, either prospectively, or retrospectively at time of pre-contrast CT angiography images on date of injury. Nor was there any evidence of pneumatocele/cyst formation. This theory is further supported by a recognized point mutation in some EDS IV patients that makes connective tissue more susceptible to laceration, as was observed in biopsied lung specimens.^7^ The patient’s pneumatocele likely caused disruption of the intrapulmonary vasculature leading to hemorrhage. Unfortunately, no autopsy information is available on this patient to support or refute our theory.

**Figure 4.**
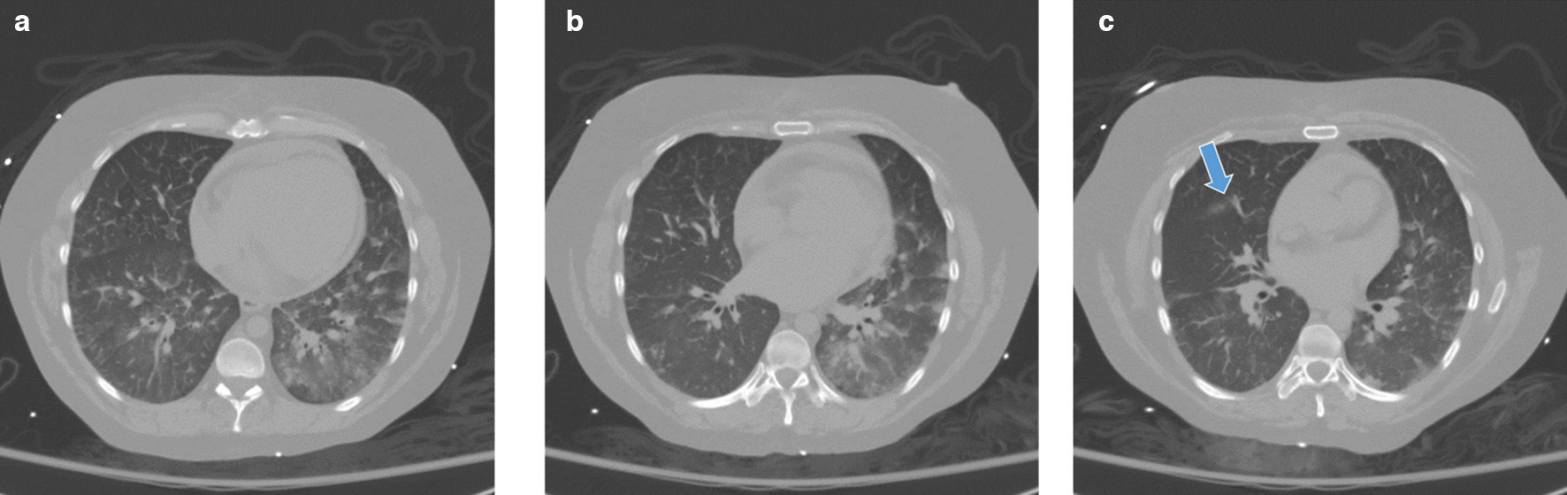
(A-C) Additional sequential imaging from the pre-contrast images taken during CT angiography on date of vascular injury. A. Axial pre-contrast imaging of the lungs near the base of the middle lobe on the date of vascular injury demonstrating no evidence of pulmonary hemorrhage involving the middle lobe prior to contrast media administration. B. Axial pre-contrast CT image a few slices more superior at the level of the proximal segmental artery branches of the middle lobe demonstrating no evidence of parenchymal hemorrhage involving the middle lobe. C. Axial pre-contrast CT imaging of the lungs more superior, now near the top of the middle lobe again demonstrating no evidence of pulmonary hemorrhage involving the middle lobe. Note the presence of the minor fissure (arrow), not to be confused for hemorrhage.

Though a rare entity, some case reports on spontaneous pulmonary hemorrhage in patients with EDS IV do exist.^8–10^ In their autopsy series of Ehlers-Danlos patients, Shields et al describe a case of spontaneous pleural/parenchymal tear of the posterior aspect of the lower lobe of the right lung in which vascular Ehlers-Danlos was diagnosed post-mortem.^8^ Kawabata et al describe pathologic evidence of pulmonary lesions related to fragility and spontaneous laceration in their series of nine patients with EDS IV.^9^ In their review of 96 cases of EDS IV, Shalhub described diagnosis of spontaneous hemothorax in 17.7% of cases.^10^Again, though we feel that that lung laceration was the underlying issue, which is in keeping with the above-mentioned reported cases, we cannot exclude a primary vascular cause, as these findings can be secondary to spontaneous rupture of vessels or of the lung parenchyma with hematoma formation and subsequent cyst formation or cavitation.^11^

Cases of spontaneous diaphragmatic rupture have been reported in the literature in EDS IV patients, a mechanism felt to be secondary to strenuous activities of the chest or activities that increase the intrathoracic or intraabdominal pressures (*e.g.* coughing, heavy breathing, emesis).^12,13^ Given this ability for spontaneous rupture of the diaphragm, a sturdier and more resilient structure relative to the lungs, it is plausible that this same phenomenon occurs with the lungs of EDS IV patients, propagating laceration and air cyst or pneumatocele formation. This theory is further supported by the fact that that these patients can form spontaneous pneumothoraces, as alluded to earlier, and was likely the cause in this case given the patient’s increased respiratory effort at time of presentation secondary to pulmonary edema.

## Teaching points

- Ehlers-Danlos Syndrome (EDS) is a rare autosomal dominant genetic condition that makes up part of the connective tissue disorders consortium. It is characterized by clinical features such as skin hyperextensibility, joint hypermobility, tissue fragility and hyperlucent skin, among other features.
- At the present, there are 13 different subtypes of EDS, with the vascular subtype (Type IV), being the most life-threatening. In type IV, type III collagen, which is used for synthesis of several types of connective tissue such as within blood vessel walls, is defective. This leads to an increased susceptibility of affected patients to undergo spontaneous vascular dissection or rupture, with potential life-threatening hemorrhage.
- Type III collagen is also used by the pulmonary fibroblasts as part of normal lung parenchyma synthesis. However, in EDS type IV patients, the defective collagen causes fragility of the lung parenchyma, which leads to easy laceration, pneumothorax or pneumatocele formation. Though the intrathoracic manifestations of EDS type IV are rare, they should not be overlooked when treating a patient with EDS who presents with suspicious intrathoracic findings. In the current case, the patient suffered a traumatic air cyst/lung laceration within a 3 min interval, which was felt to lead to formation of a large-volume pneumohemothorax.
